# Viral blood-borne infections testing and linkage to care cascade among persons who experience homelessness in the United States: a systematic review and meta-analysis

**DOI:** 10.1186/s12889-022-13786-6

**Published:** 2022-07-26

**Authors:** Ria Saha, Amanda P. Miller, Andrea Parriott, Hacsi Horvath, James G. Kahn, Mohsen Malekinejad

**Affiliations:** 1 Senior Public Health Intelligence Analyst, Medway Council, London, UK; 2 University of California, San Francisco, Philip R. Lee Institute for Health Policy Studies, 3333 California St., Ste. 265, Box 0936, San Francisco, CA 94118 USA; 3 Fielding School of Public Health, University of California, Los Angeles, Los Angeles, USA; 4 California Department of Public Health, Sacramento, CA USA; 5 Department of Epidemiology and Biostatistics, University of California, San Francisco, San Francisco, CA USA

**Keywords:** Persons who experience homelessness, Targeted testing, Care cascade, United States, HIV, HBV, HCV, Viral blood-borne infections

## Abstract

**Background:**

Persons who experience homelessness remain at increased risk for three viral blood-borne infections: human immunodeficiency virus (HIV), hepatitis B virus (HBV), and hepatitis C virus (HCV). We assessed the yield of testing and linkage to care programs targeting this population for these infections in the United States (US).

**Methods:**

We searched PubMed, Embase, Web of Science, and Cochrane Central for peer-reviewed articles through August 27, 2020. Additionally, we searched the grey literature. Two individuals independently reviewed all relevant studies to check for eligibility and extracted data for each step in the care cascade. We used random-effects model to generate weighted pooled proportions to assess yield at each step. Cumulative proportions were calculated as products of adjacent-step pooled proportions. We quantitatively synthesized data from the studies that focused on non-drug injecting individuals.

**Results:**

We identified 24 studies published between 1996–2019 conducted in 19 US states. Seventeen studies screened for HIV, 12 for HCV, and two screened for HBV. For HIV, 72% of approached were recruited, 64% had valid results, 4% tested positive, 2% were given results, and 1% were referred and attended follow-up. Of positives, 25% were referred to treatment and started care. For HCV, 69% of approached were recruited, 63% had valid results, 16% tested positive, 14% were given results, and 3% attended follow-up. Of positives, 30% were referred for treatment and 19% started care. The yield at each care cascade step differs widely by recruitment strategy (for example, for HIV: 71.6% recruited of reached under service-based with zero yield under healthcare facility-based and outreach).

**Conclusions:**

A very large proportion of this population reached for HIV and HCV care were lost in the follow-up steps and never received treatment. Future programs should examine drop-out reasons and intervene to reduce health disparities in this population.

**Supplementary Information:**

The online version contains supplementary material available at 10.1186/s12889-022-13786-6.

## Background

Persons who experience homelessness are at an increased risk for viral blood-borne infections, such as human immunodeficiency virus (HIV), hepatitis B virus (HBV), and hepatitis C virus (HCV) [1–3]. A large proportion of this population (especially youth) may engage in behaviors that place them at an increased risk for these infections, including the exchange of sex for money or food [4] and injection drug use (IDU) [5].

Due to the nature of their living conditions, persons who experience homelessness are “hard-to-reach” for important public health programs and interventions [6]. While definitions of homelessness vary, United States (US) government Code Title 42 defines “homeless individual” as “an individual who lacks housing (without regard to whether the individual is a member of a family), including an individual whose primary residence during the night is a supervised public or private facility that provides temporary living accommodations and an individual who is a resident in transitional housing” [7]. Due to methodological problems and definitional differences, size estimation of this population has remained an immense challenge. According to the National Alliance to End Homelessness, 567,715 people in the US experienced homelessness in January 2019 [8]. Other studies estimate that as many as 2.3 to 3.5 million people might experience homelessness each year in the US [9].

Persons who experience homelessness face multiple barriers to adequate health care (e.g., timely diagnosis and proper treatment) including difficulty in paying for care, lack of transportation, and lack of appropriate and timely health information [10, 11]. Based on a (2003) national survey of healthcare coverage for persons who experience homelessness, 73% of respondents reported at least one unmet healthcare need, including inability to access medical or surgical care (32%), inability to obtain medication (36%), and mental health issues (21%) [12]. Utilization appears to be particularly low among persons with certain chronic infections such as HBV and HCV [13]. This low utilization of timely healthcare among this population can severely affect their physical and mental wellbeing, which significantly increases their risk of related morbidity and mortality [14].

A companion systematic review to the present manuscript identified diverse community-based integrated tuberculosis (TB) targeted testing and treatment programs exclusively targeting persons who experience homelessness as they remain at high risk of developing both latent tuberculosis infection (LTBI) and active TB in the US [15]. The programs were exclusively designed to recruit persons who experience homelessness through different venue-based, shelter-based, or healthcare facility-based strategies with an objective to test and ensure linkage to each step of the care cascade including proportions (yield) “recruited of reached”, “valid results of recruited”, “positive of valid results”, “given results of positive”, “offered treatment” and “attended first treatment appointment of referred to treatment” (in some cases “completion of treatment” was also captured) [15]. Although a high level of variation was observed in yield (reasons partially attributed to lack of awareness, insufficient knowledge, demographic differentials, variation in recruitment methods among several others), the majority of persons experiencing homelessness across studies were successfully tested with 99.8% attending at least one session of follow-up care. Parriott (2018) also highlighted that contact investigation supplemented with community-based targeted testing and treatment programs is a high-yield and effective strategy towards sustainable TB control and elimination [16].

Identification of persons who experience homelessness with viral blood-borne infections is critical to provide timely treatment, prevent disease progression, and avert any fatal secondary infections. Since persons who experience homelessness are likely to have poor access to mainstream health care services, testing programs specifically targeting this population play an important role in public health disease control and prevention. To the best of our knowledge, this is the first systematic review to comprehensively synthesize the evidence from targeted testing and treatment programs for HIV, HCV, and HBV infections directed towards persons who experience homelessness in the US.

We aimed to systematically review the evidence to identify key variables in the outputs and outcomes (yield) of studies reporting the results of targeted testing for three viral blood-borne diseases (HIV, HCV, and HBV) among persons who experience homelessness in the US. Additionally, we also aimed to estimate proportions of these populations completing each step of the targeted testing and linkage to care cascade. Forty seven of fifty US states are not currently on track to achieve the WHO HCV elimination target by 2030. Connecticut, South Carolina, and Washington are set to achieve this target, with the remaining states set to achieve this target by 2037 [17, 18]. This work is important as it can inform gaps in coverage among persons experiencing homelessness in the US in achieving the UNAIDS 95–95-95 targets and WHO 2030 HCV elimination targets.

## Methods

We applied systematic review principles for conducting our searches and screening [19]. Specifically, we used Cochrane methods for conducting our searches and screening and followed the guidance from the Preferred Reporting Items for Systematic Reviews and Meta-analyses (PRISMA) checklist to report our findings [20]. Our protocol (Additional File 1) was registered on PROSPERO (CRD42016039432).

### Search strategies

We conducted searches in PubMed, Embase, Web of Science, and the Cochrane Central Register of Controlled Trials from the earliest records, first to 13 June 2016 and then to 27 August 2020. Our updated searches included the same databases and strategies as the first searches but covered only the period since the first searches. We initially developed a comprehensive search strategy that included multiple terms, key words, and Medical Subject Heading (MeSH) terms in PubMed related to homelessness, disease screening, viral blood-borne infections (namely for HIV, HBV, and HCV) and TB infection. We then adapted this search strategy for the other databases, adding indexing terms (e.g., Embase “Emtree” terms) where appropriate. Additional File 2 provides our detailed search strategies. Our search strategies also included the names of the 50 states of the US and other terms to enhance capture of US studies, while filtering out non-US studies.

We also searched conference abstracts from American Public Health Association annual meetings. If we saw any inconclusive abstracts from which we could not gather sufficient data, we contacted lead authors for additional information. We examined the bibliographies of our included studies and contacted experts in the field for information about any studies we may have missed. We also searched grey literature, reports published by the government, academic institutions/universities, and non-governmental organizations outside the peer-reviewed literature.

Since the original conception of this review also included studies addressing TB in addition to studies addressing HIV, HCV and HBV in persons who experience homelessness, our search strategy includes terms relevant to that condition. However, given the large number of eligible studies identified through our search and screen process, we subsequently decided to report our review of studies concerned with TB in persons who experience homelessness in the US in a separate manuscript [15].

### Eligibility criteria

We included studies reporting the results of programs (interventions) specifically targeting persons experiencing homelessness for HIV, HCV, and HBV testing in the US.

### Population

For this review, we included persons who experience homelessness as defined in the introduction. Persons who experience homelessness were those lacking a regular and adequate residence during night hours, including persons staying in homeless shelters, transitional housing, single room occupancy (SRO) facilities, structures not designed for human habitation, outdoors or temporarily staying with friends and family without plans for future stable housing. Studies that described the study populations as ‘homeless’, or used related terms such as unhoused, unstably housed, itinerant, street youth or other such terms were eligible for inclusion.

We included studies that did not intentionally recruit people who inject drugs (PWID), or if they did include PWID, reported results data stratified by IDU status. As we aimed to synthesize evidence for targeted testing of the general population of persons who experience homelessness, we decided that including only studies with non-PWID populations would better meet this objective, given the differential risk for viral blood-borne infections among PWID and non-PWID populations. We also excluded studies with a primary focus on pre-adolescent populations.

### Recruitment setting

Eligible studies used recruitment-based strategies such as service-based methods (service, homeless shelters, free meal programs), healthcare facility-based methods (community-based homeless health care clinics, or hospital emergency rooms) or outreach (through community-based strategies) to recruit study participants. Additionally, we also included studies that used hybrid approaches such as mobile testing clinics or any other service agencies that primarily serve persons who experience homelessness. We excluded studies that identified persons who experience homelessness within the context of targeted screening of other populations (e.g., PWID).

### Outcome

Eligible studies must have used a biological test such as an antibody or antigen assay to ascertain at least one of the three viral blood-borne infections (HIV, HCV, HBV). Eligible studies also needed to report, at minimum, the numbers of participants with valid biological test results and the numbers of those testing positive. We excluded studies that used only self-report to assess viral blood-borne infections, as well as studies that used stored specimens, and those reporting unlinked testing results.

### Language and study design

Studies published in any language were eligible for inclusion. We had no restrictions on study design or publication status.

### Elements of the testing and care cascade

We included and referred to the similar elements of the testing and care cascade steps (1–6) i.e., 1. “Recruited of reached” 2. “Valid results of recruited” 3. “Positive of valid results” 4. “Given results of positive” 5. “Offered treatment” and 6. “Attended first treatment appointment of referred to treatment” (in some cases there “completion of treatment” was also captured) while reporting and synthesizing the study results as mentioned in [15].

### Study screening and selection

We first merged all retrieved citations from electronic database searches into an EndNote file and removed all the duplicate records. Three review authors (APM, AP, RS) independently reviewed all the titles and abstracts of the remaining unique records to identify potentially eligible studies. They compared their respective selections at the abstract level and reached consensus through repeated in-depth discussions about potentially eligible studies. A fourth review author (MM) served as a neutral arbiter in case of lack of reviewer agreement on eligibility. Two authors (a pair from APM, AP and RS) then reviewed each full-text article deemed potentially eligible and in an identical process, made final decisions regarding study eligibility for inclusion in the review.

### Data extraction

Two review authors (APM and RS) extracted key data, including citation, study location and setting, subject recruitment venue and method, characteristics of participants, characteristics of biological tests performed, and cascade steps data, into a pre-structured data extraction spreadsheet (Additional File 3). A second review author (AP or APM) checked extracted descriptive data for accuracy, and blindly independently extracted data from the testing and linkage to care cascade. Review authors reviewed and compared these independent extractions and reconciled data via consensus. A fourth review author (MM) served as arbiter in instances where reviewer consensus could not be reached.

### Risk of bias assessment

Risk of bias was not formally assessed for this manuscript or the companion TB review [15].

### Statistical analysis and data synthesis

All statistical analyses were conducted using STATA version 14.0 (Stata Corp LP, College Station, TX, USA). We applied a random-effects meta-analytic model to generate weighted pooled proportions and 95% confidence intervals (CIs). We also assessed heterogeneity of pooled data using I^2^ and P values for the Q test [15]. We used the Wald method to calculate proportions and associated 95% CIs of persons proceeding from one step in the viral blood-borne test and linkage to care cascade to subsequent steps [15].

We calculated pooled cumulative proportions for each step of the cascade. Cumulative proportions were the products of adjacent-step pooled proportions. For instance, the cumulative proportion tested of those reached was equal to the product of the proportion recruited of those reached and the proportion tested of those recruited. Similarly, the cumulative proportion with valid test results of those reached was equal to the product of the cumulative proportion tested of those reached and the proportion with valid test results of those recruited, and so on. We also calculated cumulative proportions stratified by recruitment venue and test type for HIV and HCV but were unable to do this for HBV due to the limited number of identified studies. CIs for the cumulative proportions were calculated using a simulation method that was described in detail in our prior published review [15]. Data were insufficient to perform additional proposed data synthesis focused exclusively on persons who experience homelessness and use injection drugs.

## Results

### Results of the searches

**Fig. 1:**
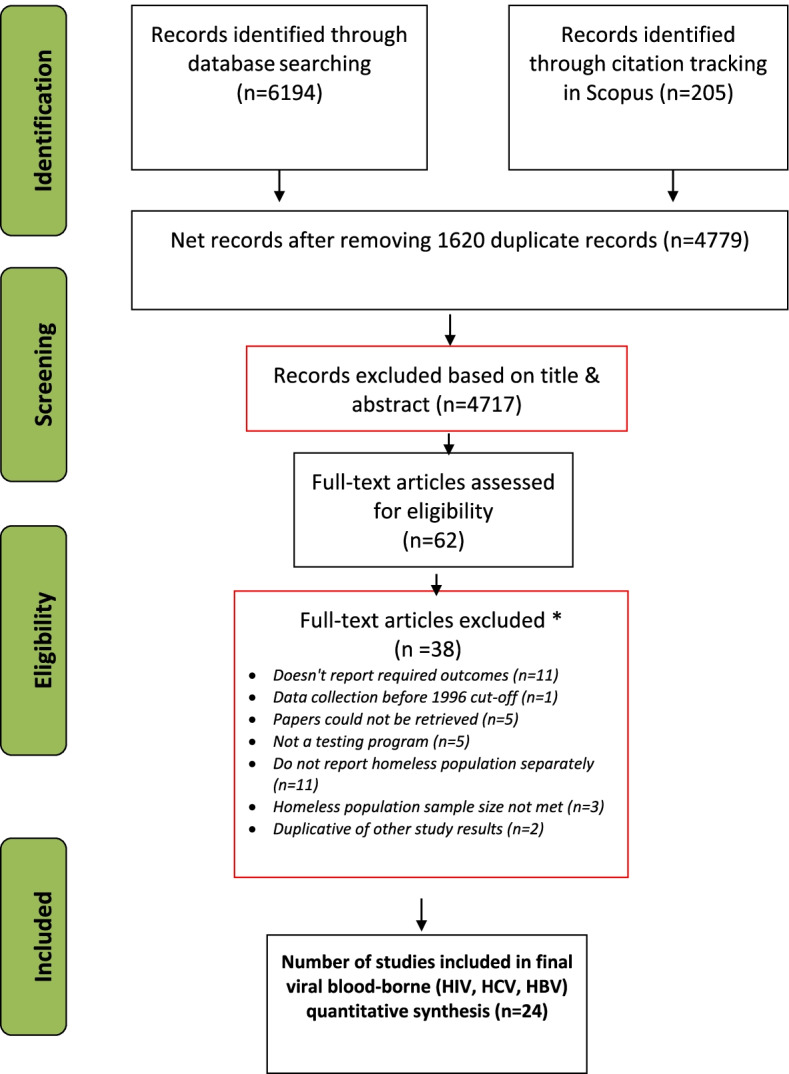
PRISMA flowchart. Process for identifying eligible studies of viral blood-borne infections targeted testing and linkage to care among persons who experience homelessness in the US

We performed an initial search of the literature in 2016, and then updated the searches in August 2020. We retrieved a total of 4636 citations, of which 24 studies (including three conference abstracts) met inclusion criteria. Of these, 22 were observational, with the majority being single arm cohort (*N* = 9) and cross-sectional (*N* = 9) (Table 1). Two were retrospective studies and two were randomized controlled trials (RCTs). The design of two studies was unclear. Figure 1 illustrates our process for identification and screening of citations. Additional file 4 provides more detail on the article screening process at the full text level. Table 1 provides a description of the included studies.

**Table 1:** Characteristics of 24 targeted testing and linkage to care studies of viral blood-borne infections among persons who experience homelessness in the US

Study	Setting	Study design	Recruitment method	Target population	Data collection	Test (specimen) by disease		
						**HBV**	**HCV**	**HIV**
Anaya 2010 [22]	Los Angeles, CA	Double-Arm cohort	Shelter-based	VA-eligible persons who experience homelessness	2006–2007	–	–	Rapid-Ab or Ab (mixed)
Anaya 2015 [23]	Los Angeles, CA	Single-Arm cohort	Shelter-based	Adults who experience homelessness	2009–2011	–	–	Rapid-Ab + conf (oral fluid)
Bell 2003 [24]	New York, City, NY	Single-Arm cohort	Outreach	Youth who experience homelessness	1998–2000	–	–	Ab (blood)
Benitez 2020 [25]	Los Angeles, CA	Retrospective study	Healthcare facility-based (Los Angeles Christian Health Centers)	Predominantly persons who experience homelessness including both unsheltered persons and those who reside in supervised shelters or transitional housing at night who are at risk for HIV, drug use	2016–2019	–	Non-rapid Ab (blood)	–
Bowles 2008 [26]	Boston, MA; Chicago, IL; Washington, DC; Kansas City, MO; Detroit, MI	Single-Arm cohort	Shelter-based	HIV-unaware adults who experience homelessness	2004–2006	–	–	Rapid-Ab (mixed)
Boyce 2009 [27]	Honolulu County, HI	Cross-sectional	Shelter-based	Adults who experience homelessness	2006–2006	HBsAg (blood)	Ab (blood)	–
Bucher 2007 [28]	San Francisco, CA	Single-Arm cohort	SROs, shelters, and free meal programs	Persons who experience homelessness & unstably housed adults	2003–2004	–	–	Rapid-Ab + conf (oral fluid)
Caton 2013 [29]	New York City, NY	Cross-sectional	Shelter-based	Women who experience homelessness	2007–2008	–	–	Ab (oral fluid)
Gelberg 2012 [30]	Los Angeles, CA	Single-Arm cohort	Shelters and meal programs	Adults who experience homelessness	2003–2004	–	Ab (blood)	Ab (blood)
Fuster & Gelberg 2019 [14]	Los Angeles, CA	Single-Arm cohort	Other venue based (service programs, shelter programs, & meal programs)	Participants who tested seropositive for HIV/HCV/HBV from the baseline sample	2003–2004 (9 months)	Rapid Ab (blood)	Rapid Ab (blood)	Ag detection (blood)
Grimley 2006 [21]	Alabama	Cross-sectional	Shelter-based	Adults who experience homelessness	–	–	–	Ab (oral fluid)
Hall 2004 [31]	San Francisco, CA	Single-Arm cohort	SROs, facilities maintaining 73% of shelter beds and venues providing 88% of free lunches in the city	Unstably housed adults with HIV	1996–2000	–	Ab (blood)	–
Hooshyar 2014 [32]	Dallas, Texoma, and Fort Worth, TX	Cross-sectional	Outreach	VA-eligible adults who experience homelessness	2011–2011	–	–	Rapid-Ab (NR)
Khalili 2019 [33]	San Francisco, CA; Minneapolis, MN	Single-Arm cohort	Shelter-based	Clients who experience homelessness in homeless shelters	Not reported (conference abstract)	–	Rapid Ab (blood)	–
Klinkenberg 2003 [5]	St. Louis, MO	Unclear	Outreach and referral (hospitals, social service agencies, shelters, soup kitchens, street)	Adults who experience homelessness with co-occurring severe mental illness and substance use disorders	2000–2000	–	Ab (blood)	Ab (mixed)
Magura 2000 [34]	New York City, NY	Unclear, possibly mixed	Soup kitchens	Soup kitchen guests	1997–1997	HBsAg (blood)	–	Ab (mixed)
Page 2017 [35]	San Francisco, CA	Cross-sectional	Outreach (free meal programs, homeless shelters, and low-cost single room occupancy hotels)	Persons who experience homelessness and unstably housed adult women	2008–2010	–	Ag detection (blood)	Ag detection (blood)
Preston 2016 [36]	New Orleans, LA	Single-Arm cohort	Shelter-based	Adults who experience homelessness	Not reported (conference abstract)	–	Rapid-Ab (NR)	–
Robbins 2010 [37]	San Francisco, CA	Cross-sectional	Shelters and "targeted" sampling, which is not defined in the paper	Adult who experiences homelessness PWID	2003–2005	–	–	Ab (mixed)
Rosenblum 2001 [38]	New York City, NY	Cross-sectional	Healthcare facility-based	Adults who experience homelessness	1997–1998	–	Ab (blood)	Rapid-Ab or Ab (NR)
Schwarz 2008 [39]	Baltimore, MD	Single-Arm cohort	Primarily shelters (11% recruited from soup kitchens and adult IDU clinics.)	Parents with children who experience homelessness	2001–2004	–	Ab + Ag (blood)	–
Sena 2016 [40]	Durham, NC	Cross-sectional	Healthcare facility-based	Adults with HCV risk	2012–2014	–	Ab + NAAT (blood)	–
Stewart 2020 [41]	North Seattle, WA	Retrospective abstraction of electronic medical record data	Healthcare facility-based (SHE clinic)	Women with unstable housing (i.e.; experiencing homelessness) who reported exchanging sex for money or nonmonetary items and who inject drugs	2018 (4 months)	–	–	NA detection (endocervical swab samples)
Tsu 2002 [42]	Portland, OR	Double-Arm cohort	Outreach	Youth with HIV risk	1998–1999	–	–	Ab (oral fluid)

***Notes*: *Ab*** antibody, ***Ag*** antigen, ***conf*** confirmation, ***HBsAg*** Hepatitis B surface antigen, ***IDU*** injection drug user, ***NA*** not Applicable, ***NR*** not reported, ***PWID*** people who inject drugs, ***SHE* clinic:** safe. Healthy. Empowered clinic, ***SRO*** single resident occupancy, ***VA*** Veterans Administration

Studies that met inclusion criteria were published between 2002 and 2020; data collection periods ranged from 1996 to 2019. Studies were conducted in 19 US states. Ten were conducted in California, four were conducted in New York and the remainder were conducted in other states. Most studies reported specific study locations, but one [21], conducted in Alabama, did not specify the city or county. The majority of included studies (*N* = 21) were conducted at a single site; three had results from multiple sites. Studies primarily targeted all persons experiencing homelessness, but some studies focused on specific sub-population experiencing homelessness such as youth, women, persons with co-occurring severe mental illness and substance use disorder, Veterans Administration (VA)-eligible populations, and PWID. Fifteen studies recruited participants via programs providing services such as shelter and meals, while five studies involved other recruitment approaches such as outreach. Four studies recruited targeted populations from healthcare facilities. Seventeen studies screened for HIV, twelve screened for HCV, and two screened for HBV. Studies were diverse in respect to recruitment methods, specimens collected for diagnosis/screening and types of targeted laboratory tests used to ascertain viral blood-borne infection.

### Viral blood-borne infections testing and linkage to care cascade

We generated cascade estimates, which were derived from the pooled proportions of the studies presented in Table 2. Estimates indicate the proportions of participants from the preceding step that complete a given step.

**Table 2:** Pooled proportions (presented in %) and 95% CIs for steps in targeted testing and linkage to care cascade by infection type: persons who experience homelessness in the US

Disease type	Recruited of Reached	Valid test Results of recruited	Test ( +) of valid test results	Given ( +) Results of test ( +)	Referred to follow-up of given ( +) results	Attended follow-up of referred to follow-up
HIV	71.6% (67.5%,75.4%) Four studies	89.9% (80.1%,96.8%) Eight studies	5.8% (2.2%, 11.0%) 16 studies	50.8% (0.3%,99.9%) Three studies	68.3% (29.4%,97.9%) Two studies	100% (20.6%,100%) One study
HCV	68.8% (23.7%,98.5%) Three studies	94.8% (81.9%,100%) Seven studies	26% (15.6%,37.9%) 12 studies	84.5% (81.0%,87.7%) Two studies	100% (99%,100%) One study	24.4% (20.7%,28.4%) Two studies

*Note*: Since none of the studies on HBV mention stratification by IDU sub-populations we excluded those from our final quantitative data reporting

For the HIV test and treat cascade, our pooled estimates suggest 71.6% (95% CI 67.5, 75.4%) of those reached would be recruited; 89.9% (95% CI 80.1%, 96.8%) of those tested would receive valid test results; 5.8% (95% CI 2.20%, 11.0%) with valid results would test positive; 50.8% (95% CI 0.3%, 99.9%) of those who tested positive would be given results; 68.3% (95% CI 29.4%, 97.9%) of those positive would be referred to follow-up for treatment; and 100% (95% CI 20.6%, 100%) of those referred would attend follow-up.

For the HCV test and treat cascade, our pooled estimates suggest that 68.8% (95% CI 23.7%, 98.5%) of those reached would be recruited; 94.8% (95% CI 81.9%,100%) of those tested would receive valid test results; 26% (95% CI 15.6%, 37.9%) of those with valid results would test positive; 84.5% (95% CI 81.0%, 87.7%) of who those tested positive would be given results; 100% of those positive would be referred to follow-up for treatment (95% CI 99%, 100%) and 24.4% (95% CI 20.7%, 28.4%) of those referred would actually attend follow-up.

A pooled cascade was not estimated for HBV because studies reporting on this cascade did not stratify results by IDU status. For all pooled proportions under different cascade steps, the proportion of variability in the effect estimates that is due to heterogeneity (I^2^) was greater than 75% where I^2^ was measurable. Except for “recruited of reached” under HIV (I^2^ = 69.7%), for which pooled proportion of all studies was 71.6%.

**Fig. 2:**
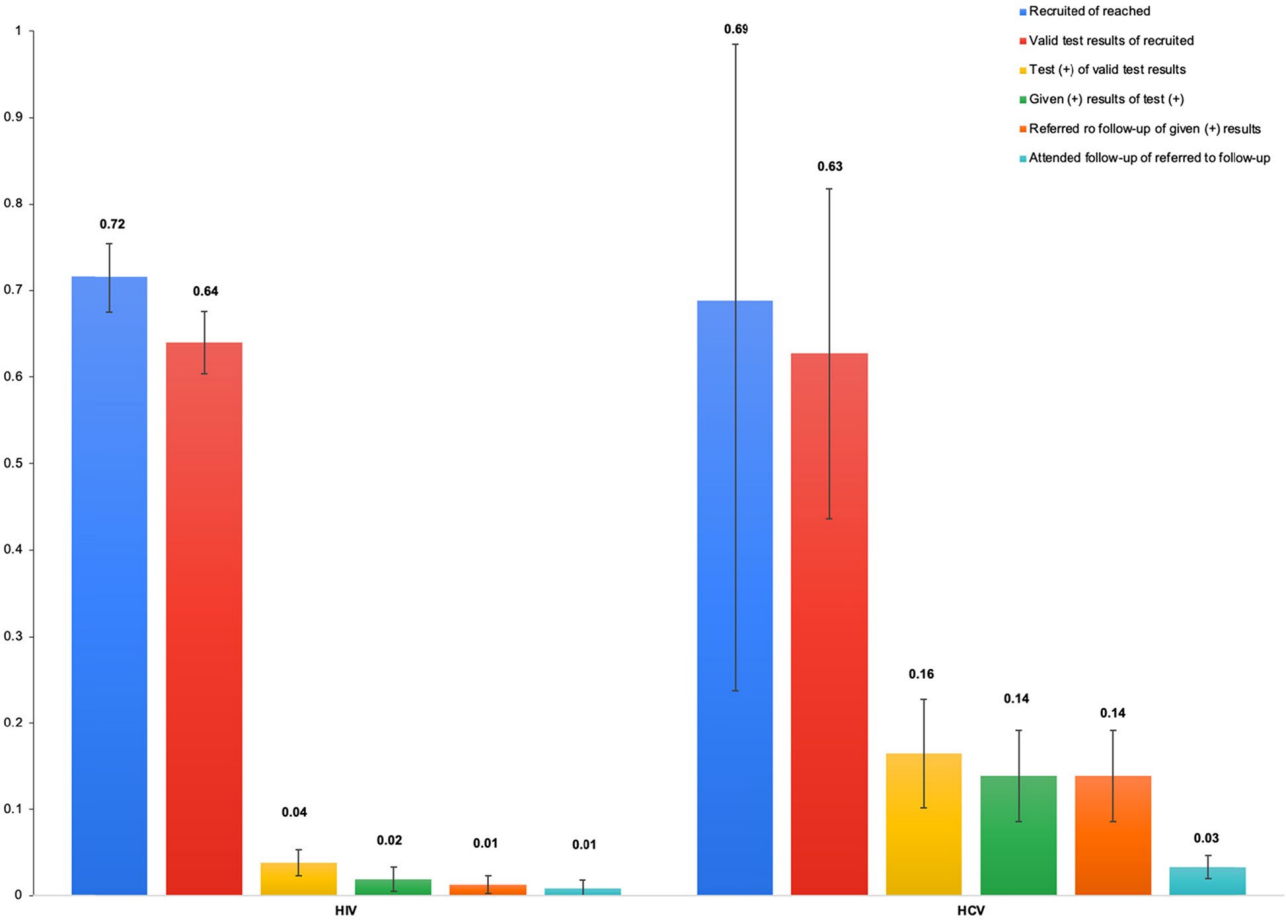
Cumulative proportions of participants retained by cascade step via viral blood-borne infections HIV and HCV targeted testing and linkage to care among persons who experience homelessness in the US

We also calculated cumulative proportions of participants retained by each cascade step via viral blood-borne infections targeted testing and linkage to care among persons who experience homelessness in the US (Fig. 2).

For a hypothetical group of 100 participants reached for HIV screening, we estimate that 72 (95% CI 67.5, 75.4) would be recruited, 64 (95% CI 56.6, 70.6) would receive valid test results; 4 (95% CI 1.3, 7.1) would test positive; 2 (95% CI 0, 5.1) would be given results; 1 (95% CI 0, 3.8) would be referred to follow-up for treatment and 1 (95% CI 0, 3.2) would attend follow-up. Notably, of those who tested positive for HIV, 25% were referred to treatment and attended follow-up visits.

From Fig. 2, the cumulative proportions for referred to follow-up and those who attended follow-up are similar, due to low values and wide uncertainty (Table 2).

For HCV, in a hypothetical group of 100 participants reached, we estimate that 69 (95% CI 23.7, 98.5) would be recruited, 63 (95% CI 22.1, 93.9) would receive valid test results; 16 (95% CI 5.2, 29.3) would test positive; 14 (95% CI 4.4, 24.8) would be given results; 14 (95% CI 4.4, 24.7) would be referred to follow-up for treatment and 3 (95% CI 1.0, 6.1) would attend follow-up. The cumulative proportions for the given results and referred to follow-up were similar, due to low values and wide uncertainty (Table 2). Of those who tested positive for HCV, 30% were referred for treatment and 19% attended follow-up visit.

### HIV and HCV cascade steps by recruitment and testing type sub-analyses

We explored whether two implementation characteristics of HIV and HCV programs, recruitment site (Tables 3 and 4) and testing type (Tables 5 and 6), might affect participants’ retention in three important cascade steps: 1) valid results received by recruited subjects; 2) proportion testing positive of those with valid results; and 3) proportion who received results of those testing positive. Under HIV, the pooled proportions of participants retained in those cascade steps differed by recruitment site/type in two cases. Healthcare facility-based testing and testing in other service-based approaches significantly differed in the proportions of recruited participants receiving valid test results (100% in one study versus 89.7% in six studies; *p* < 0.0001). The proportions of participants who tested HIV positive of those with valid results differed significantly across healthcare facility-based settings, other service-based settings, and outreach (20.7% in one study versus 6.8% in eleven studies versus 0.8% in three studies; *p* < 0.0001). For HCV, the proportion of valid results of recruited participants differed significantly across healthcare facility-based recruitment method and other service-based methods (100% in one study versus 97.6% in five studies; *p* < 0.0001) and across all three categories for the proportions who tested HCV positive of those with valid results (28.6% in two studies versus 25.3% in nine studies versus 29% in one study; *p* < 0.0001).

**Table 3:** Proportions (%) for select steps in HIV targeted testing cascade among persons who experience homelessness, by recruitment venue/type

HIV cascade: Healthcare facility-based				
**Cascade steps**	**Study**	**N**	**Proportion**	**95% CI**
Recruited of reached	No studies	-	-	-
Valid results of recruited	Rosenblum 2001 [38]	139	100%	97.3%,100%
Positive of valid results	Rosenblum 2001 [38]	21	15.1%	10.1%, 22%
Given results of positive	No studies	-	-	
Referred to treatment of given results	No studies	-	-	-
Attended first treatment appointment of referred to treatment	No studies	-	-	-
**HIV cascade: Other service-based**				
**Cascade steps**	**Study**	**N**	**Proportion**	**95% CI**
Recruited of reached	Anaya 2010 [22]; Bucher 2007 [28]; Grimley 2006 [21]; Magura 2000 [34]	1825	71.6%	67.5%, 75.4%
Valid results of recruited	Anaya 2010 [22]; Bucher 2007 [28]; Grimley 2006 [21]; Magura 2000 [34]; Page 2017 [35]; Stewart 2020 [41]	2024	89.7%	74.9%, 96.6%
Positive of valid results	Anaya 2010 [22]; Anaya 2015 [23]; Bowles 2008 [26]; Bucher 2007 [28]; Caton 2015 [29]; Gelberg 2012 [30]; Gelberg 2019 [14]; Grimley 2006 [21]; Magura 2000 [34]; Page 2017 [35]; Stewart 2020 [41]	403	6.8%	2.1%, 13.9%
Given results of positive	Anaya 2015 [23]; Grimley 2006 [21]	7	81.2%	43.3%, 100%
Referred to treatment of given results	Anaya 2015 [23]; Grimley 2006 [21]	6	92.5%	46.9%, 100%
Attended first treatment appointment of referred to treatment	Grimley 2006 [21]	1	100%	20.7%, 100%
**HIV cascade: Outreach**				
**Cascade steps**	**Study**	**N**	**Proportion**	**95% CI**
Recruited of reached	No studies	-	-	-
Valid results of recruited	No studies	-	-	-
Positive of valid results	Bell 2003 [24]; Hooshyar 2014 [32]; Tsu 2002 [42]	5	0.8%	0%, 3.8%
Given results of positive	Tsu [42]2002	0	-	-
Referred to treatment of given results	No studies	-	-	-
Attended first treatment appointment of referred to treatment	No studies	-	-	-

**Table 4:** Proportions for select steps in HCV targeted testing cascade among persons who experience homelessness, by recruitment venue/type

HCV cascade: Healthcare facility-based				
**Cascade steps**	**Study**	**N**	**Proportion**	**95% CI**
Recruited of reached	No studies	-	-	-
Valid results of recruited	Rosenblum 2001[38]	167	100%	97.3%,100%
Positive of valid results	Sena 2016 [40]; Rosenblum 2001[38]	88	28.6%	22.8%,34.7%
Given results of positive	No studies	-	-	-
Referred to treatment of given results	No studies	-	-	-
Attended first treatment appointment of referred to treatment	No studies	-	-	-
**HCV cascade: Other service-based**				
**Cascade steps**	**Study**	**N**	**Proportion**	**95% CI**
Recruited of reached	Gelberg 2012 [30]; Preston 2016 [36]; Hall 2004 [31]	1168	69%	23.7%,98.6%
Valid results of recruited	Gelberg 2012 [30]; Preston 2016 [36]; Schwarz 2008 [39]; Hall 2004 [31]; Boyce 2009 [27]	1508	97.6%	91.4%,100%
Positive of valid results	Gelberg 2012 [30]; Preston 2016 [36]; Schwarz 2008 [39]; Hall 2004 [31]; Boyce 2009 [27]; Page 2017 [35]; Benitez 2019[]; Khalili 2019 [33]; Gelberg 2019 [14]	1508	25.3%	13.4%,39.3%
Given results of positive	Swarchtz 2008 [39]; Benitez 2019[]	399	84.5%	81%,87.7%
Referred to treatment of given results	Benitez 2019[]	375	100%	99.5%,100%
Attended first treatment appointment of referred to treatment	No studies	-	-	-
**HCV cascade: Outreach**				
**Cascade steps**	**Study**	**N**	**Proportion**	**95% CI**
Recruited of reached	No studies[]	-	-	-
Valid results of recruited	No studies	-	-	-
Positive of valid results	Klinkenberg 2003[5]	34	29%	-
Given results of positive	No studies	-	-	-
Referred to treatment of given results	No studies	-	-	-
Attended first treatment appointment of referred to treatment	No studies	-	-	-

**Table 5:** Proportions for select steps in HCV targeted testing cascade among persons who experience homelessness, by test type

Rapid Ab			
**Cascade steps**	**Study**	**Proportion**	**95% CI**
Recruited of reached	Preston 2016 [36]	25%	22%,28.2%
Valid results of recruited	Preston 2016 [36]	100%	98%,100%
Positive of valid results	Preston 2016 [36]; Khalili 2019 [33]	21.3%	18.7%,23.9%
Given results of positive	No studies	-	-
Referred to treatment of given results	Preston 2016 [36]; Khalili 2019 [33]	72.4%	66%,78.5%
Attended first treatment appointment of referred to treatment	Khalili 2019 [33]	61.7%	52.2%,70.3%
**Non-rapid Ab**			
**Cascade steps**	**Study**	**Proportion**	**95% CI**
Recruited of reached	Gelberg 2012 [30]; Hall 2004 [31]	72.7%	71%,75.2%
Valid results of recruited	Gelberg 2012 [30]; Hall 2004 [31]; Klinkenberg 2003 [5]; Schwarz 2008 [39]; Rosenblum 2001 [38]; Boyce 2009 [27]	93.4%	76.6%,100%
Positive of valid results	Gelberg 2012 [30]; Klinkenberg 2003 [5]; Hall 2004 [31]; Schwarz 2008 [39]; Rosenblum 2001 [38]; Boyce 2009 [27]; Sena 2016 [40]; Page 2017 [35]; Benitez 2019[]; Gelberg 2019 [14]	26.9%	14.1%,42%
Given results of positive	Schwarz 2008 [39]; Benitez 2019[]	84.5%	81%,87.7%
Referred to treatment of given results	Benitez 2019[]	84.7%	81%,87.7%
Attended first treatment appointment of referred to treatment	Benitez 2019[]	15.7%	12.4%,19.8%

**Table 6:** Proportions for select steps in HIV targeted testing cascade among persons who experience homelessness, by test type

Rapid Ab			
**Cascade steps**	**Study**	**Proportion**	**95% CI**
Recruited of reached	Bucher 2007 [27]	75.2%	73%,77.2%
Valid results of recruited	Bucher 2007 [27]	99.3%	98.6%,99.6%
Positive of valid results	Hooshyar 2014 [32]; Bucher 2007 [27]; Bowles 2008 [26]	3.4%	0%,16.6%
Given results of positive	No studies	-	-
Referred to treatment of given results	No studies	-	-
Attended first treatment appointment of referred to treatment	No studies	-	-
**Non-rapid Ab**			
**Cascade steps**	**Study**	**Proportion**	**95% CI**
Recruited of reached	Grimley 2006 [21]; Magura 2000 [34]	69%	65.7%,72.3%
Valid results of recruited	Grimley 2006 [21]; Klinkenberg 2003 [5]; Magura 2000 [34]; Page 2017 [35]; Stewart 2020 [41]	86.4%	79.3%,92.3%
Positive of valid results	Grimley 2006 [21]; Gelberg 2012 [14]; Caton 2015 [29]; Bell 2003 [24]; Klinkenberg 2003 [5]; Magura 2000 [34]; Tsu 2002 [42]; Page 2017 [35]; Stewart 2020 [41]; Gelberg 2019 [14]	7.3%	1.9%,15.8%
Given results of positive	Grimley 2006 [21]; Tsu 2002 [42]	18%	0%,77.2%
Referred to treatment of given results	Grimley 2006 [21]	50%	9.5%,90.5%
Attended first treatment appointment of referred to treatment	Grimley 2006 [21]	100%	20.7%,100%
**Unknown**			
**Cascade steps**	**Study**	**Proportion**	**95% CI**
Recruited of reached	Anaya 2010 [22]	71.3%	63.2%,78.3%
Valid results of recruited	Anaya 2010 [22]; Rosenblum 2001 [38]	93.6%	90%,96.4%
Positive of valid results	Anaya 2015 [23]; Anaya 2010 [22]; Rosenblum 2001 [38]	4.2%	0%,16.2%
Given results of positive	Anaya 2015 [23]	85.7%	48.7%,97.4%
Referred to treatment of given results	Anaya 2015 [23]	71.4%	35.9%,91.8%
Attended first treatment appointment of referred to treatment	No studies	-	-

### Comparison of cascade data by rapid-antibody testing versus non-rapid testing

In (Tables 5 and 6), cascade data for HCV and HIV studies are reported by test type, with results stratified into three categories: rapid antibody testing, non-rapid testing, and test type unknown. In HCV studies that provided rapid testing, the proportion of participants who were recruited of those who were reached, had valid test results of those who were recruited, and had positive results of those with valid results were 25%, 100%, and 21.3%, respectively. Among those who were linked to care, 72% were referred to treatment and 62% attended the first appointment of those referred to treatment. For studies with non-rapid testing, estimates were 73%, 93%, 27%, 84% for the proportion of participants who were recruited of those who were reached, had valid test results of those who were recruited, had positive results of those with valid results and were given results of positive, respectively. The proportions under valid results of recruited and positive of valid results differed significantly across these two testing types (*p* < 0.0001). Under linkage to care, 85% were referred to treatment and 16% attended the first appointment of the referred treatment. For HIV studies that provided rapid testing, the proportions of participants who were recruited of those who were reached, had valid test results of those who were recruited, and had positive results of those with valid results, were 75%, 99%, and 3%, respectively (Table 5). For the studies using non-rapid testing, these estimates were 69%, 86%, 7% and 18% for the proportions of participants who were recruited of those who were reached, had valid test results of those who were recruited, had positive results of those with valid results and given results of positive, respectively. For the linkage to care cascade, 50% were referred to treatment of given results and 100% attended first treatment appointment.

## Discussion

### Strategies to improve yield in HIV testing cascade

Our systematic review used comprehensive searches and Cochrane review methods to identify reports of studies describing the yield of targeted HIV, HCV, and HBV testing and linkage to care cascades among persons who experience homelessness in the US. We used rigorous meta-analytic and other statistical methods to estimate the proportion of individuals who were retained at each step of the testing and linkage-to-care cascade. The 24 identified studies, of which 22 were observational or descriptive, provide provisional evidence suggesting benefit of such integrated targeted programs. Overall, targeted testing may be an effective strategy for linking persons who experience homelessness with serious viral blood-borne infections to care, despite dropouts at the recruitment step (Tables 3 and 4,Tables 5 and 6). For both HIV and HCV targeted testing cascades, other service-based methods had the best yield relative to other recruitment strategies (Tables 3 and 4).

For HCV, yield across the cascade was better in studies using non-rapid testing compared to those using rapid testing, especially where there were significant dropouts at the recruitment stage under rapid testing (Table 5). A significantly higher proportion of participants had positive results under non-rapid testing (27%) compared with rapid testing (21.3%). This might be explained by the diverse recruitment strategies (other service-based [shelters/shelter beds, SROs, meal programs, free lunch venues in the city]; health-care facility based; and outreach programs) compared to rapid testing where only service-based strategies (shelter-based) were used (Table 4). Recruiting high-risk populations from diverse places increases the representativeness of this population, and thus increases the probability of identifying and recruiting those at highest risk as some shelter homes don’t permit PWID. Differences in retention and positivity between test types in the HIV cascade were not as pronounced for the early steps in the cascade. However, the results should be interpreted cautiously given the lack of data across numerous HIV cascade steps under rapid testing (Table 6) and the non-randomized nature of most of our review’s evidence, as well as remaining gaps in the evidence for many cascade steps.

Overall, from Fig. 2 it can be interpreted that linkage to care for both HIV and HCV, was poor, with significant dropouts throughout the care cascade. Findings can be used to guide improvements in uptake of testing and retention. Although not our focus, two of the included studies [22, 42] reported RCT data on the effectiveness of strategies to improve uptake of HIV testing and receipt of results in this population. Anaya and colleagues (2010) compared the effect of provision of on-site rapid testing versus referral to a VA clinic for off-site testing among 97 veterans experiencing homelessness in Los Angeles, California [22]. They found that those who were recruited and offered onsite testing were 30 times (*p* < 0.0001) more likely to be tested than those receiving a referral [22]. Nearly all (99%) of those offered onsite testing received their test results, versus no participants in the referral group [22]. These results are consistent with results reported by Khalili and colleagues (2019) (although an observational non-randomized cohort/follow-up study), where onsite testing was reported along with implementation of formal HCV education, were factors identified as enhancing HCV testing [34]. Additionally, receipt of formal HCV education was identified as a significant factor (along with existing factors in the program implementation structure) to enhance willingness to received therapy (85% were willing post education; *p* < 0.01) and adherence (63%) to achieve an end of therapy response (73%) [33]. In another RCT, Tsu and colleagues (2002) compared the effect of delivering HIV test results face-to-face versus offering the option of receiving results by phone or face-to-face among 351 youths who experience homelessness in Portland, Oregon, and found that for this population the option of getting results by phone improved receiving test results by 60% (*p* < 0.0001) [42]. The two included RCTs [22, 42] focused exclusively on testing and linkage to care in the context of HIV testing, so it is unclear how effective such strategies would be in programs seeking to engage persons who experience homelessness for HCV or HBV testing programs. Such strategies should be explored for the HCV and HBV test and treat programs.

Our review had several limitations. First, our searches had two vulnerabilities. Our use of a filter designed to exclude non-US studies may have inadvertently omitted eligible studies. In limiting the search frame of our updated searches to 2016–2020, it is also possible that we missed eligible older studies added to the databases after our searches. The second limitation is that all but two of the studies we identified were observational or descriptive in design. Although we did not formally assess the risk of bias in each study, it is well-established that the results of non-randomized studies must be interpreted conservatively, especially when the data from such studies are pooled [19]. We can speculate on the types of biases that may have affected our results. Publication bias is important. The results of targeted testing and treatment programs for persons who experience homelessness are rarely published because assessments performed by busy public health workers are intended primarily to inform local program management. It is possible that programs that publish differ from programs that do not. For example, programs may publish about exceptionally successful achievements, or conversely, about programs that faced exceptional challenges. Another consequence of incomplete reporting of targeted testing programs is that it is impossible to know what proportion of persons experiencing homelessness are currently reached by these programs, which in turn makes it difficult to estimate the possible effects of scaling up testing in this population. Misclassification is another potential source of bias; numbers of persons who were retained at each step of the cascade may not have been recorded correctly, and mistakes may have been made in interpreting test results. Studies did not generally provide sufficient evidence to evaluate this risk of bias, though the random effects model that we used to calculate the pooled proportions found high heterogeneity (> I^2^ 75) in many of our pooled proportions, which potentially signifies wide inter-study variation of different programmatic factors in the included studies rather than random variability. This probably also affects the proportions retained across all cascade steps. Therefore, while interpreting the results from our study it is important to consider the CIs along with the pooled proportions.

Given the absence of evidence for the healthcare facility-based recruitment strategy (HIV: *N* = 1; HCV: *N* = 2) and insufficient data for outreach-based targeted programs (HIV: *N* = 3; HCV: *N* = 1), it is not possible to conclude the comparative efficacy across recruitment strategies to improve linkage to care. Similarly, missing data throughout the cascade precludes drawing conclusions regarding comparative efficacy by test types. Since most of the persons who experience homelessness do not have adequate access to services/organized testing programs it is possible that our included studies in the review missed the majority of this population without access to testing and related integrated programs. Also, since we only included studies which stratify by PWID (so we could focus on non-PWID population groups) in our quantitative synthesis and reporting, we were unable to quantitatively report results for HBV studies (*N* = 2).

Our review also has important strengths. We followed Cochrane guidance in our search and screening process. We used rigorous, customized statistical methods to estimate the proportions of participants retained at each step in the cascade of care. These estimates represent an important synthesis of available, if imperfect, evidence for this critical public health management issue.

## Conclusions

Overall, a very large proportions of the targeted population who were reached for HIV and HCV care were lost to follow-up in subsequent care cascade steps. Future targeted testing and treatment programs should investigate drop-out reasons and intervene to improve retention. Most included studies used an assortment of service-based recruitment strategies, while outreach was the most underutilized strategy. Since other service-based recruitment strategies may not reach the entire population experiencing homelessness, future studies should consider involving outreach as a targeted mechanism of screening and testing. Our work provides valuable estimates and insights in understanding the progress towards the elimination of HIV and HCV within high-risk population as targeted by UNAIDS and WHO. Recognizing the disproportionate burden of viral blood-borne infections in high-risk populations, establishing effective screening programs integrated with accessible care is imperative to achieve rapid reduction and elimination of these diseases.

## Supplementary Information

**Additional file 1.** 

**Additional file 2.**

**Additional file 3.**

## Data Availability

All data generated or analyzed during this study are included in this published article and its additional files 1,2,3, and 4.

## Abbreviations

TB: Tuberculosis
LTBI: Latent tuberculosis infection
HIV: Human immunodeficiency virus
HBV: Hepatitis B virus
HCV: Hepatitis C virus
SRO: Single room occupancy
Ab: Antibody
Ag: Antigen
HBsAg: Hepatitis B surface antigen
IDU: Injection drug user
PWID: People who inject drugs
RCT: Randomized controlled trial
US: United States
PRISMA: Preferred Reporting Items for Systematic Reviews and Meta-analyses
