# Supplementary material for: Viral blood-borne infections testing and linkage to care cascade among persons who experience homelessness in the United States: a systematic review and meta-analysis
Source: BMC Public Health. 2022 Jul 26;22:1421. doi: 10.1186/s12889-022-13786-6 (PMC9327172; doi:10.1186/s12889-022-13786-6)
Supplement: Supplementary file 1 — Additional file 1. [file 12889_2022_13786_MOESM1_ESM.docx]

# Additional file 2

# Database search strategies and yield

# Search dates:

# 13 June 2016 (earliest records to search date)

# 27 August 2020 (June 2016 to search date)

Searched databases: PubMed, Embase, Web of Science, Cochrane CENTRAL

No date or language limits.

Resulting records:

| **Database** | **Yield, earliest records to 2016** | **Yield, 2016-2020** | **Total yield** |
| --- | --- | --- | --- |
| PubMed | 1,033 | 283 | 1,316 |
| Embase | 1,477 | 1,783 | 3,260 |
| Web of Science | 1,000 | 509 | 1,509 |
| Cochrane CENTRAL | 56 | 53 | 109 |
| ALL DATABASES | 3,566 | 2,628 | **6,194** |

- **Total records: k = 6,194**
- **Duplicates removed: k = 1,446**
- **Records remaining: k = 4,748**

| **Search** | **PubMed query** | **Records up to 2016** | **Records 2016-2020** | **TOTAL RECORDS** |
| --- | --- | --- | --- | --- |
| **#5** | **Search #1 AND #2 AND #3 AND #4** | 1033 | 283 | **1316** |
| #4 | Search (Tuberculosis[mh] OR HIV infections[mh] OR hepatitis b[mh] OR hepatitis c[mh] OR tuberculosis[tiab] OR TB[tiab] OR LTBI[tiab] OR HIV*[tiab] OR "human immunodeficiency virus"[tiab] OR "blood-borne”[tiab] OR bloodborne[tiab] OR “hepatitis b”[tiab] OR “hepatitis c”[tiab] OR HBV[tiab] OR HCV[tiab] OR “hep b”[tiab] OR “hep c”[tiab]) | 671627 | 119572 | 791199 |
| [#3](http://www-ncbi-nlm-nih-gov.ucsf.idm.oclc.org/pubmed/advanced) | Search (“Mass Screening”[mh] OR "Mobile Health Units"[Mesh] OR screen*[tiab] OR testing[tiab] OR program*[tiab] OR community-based[tiab] OR outreach[tiab] OR recruit*[tiab]) OR (target*[tiab] AND test*[tiab]) OR (peer[tiab] OR peer-based[tiab] OR chain-referral[tiab] OR snowball[tiab] OR sampling[tiab] OR time-location[tiab] OR respondent-driven[tiab] OR “social marketing” [tiab] OR “health fair” [tiab]) OR (engage[tiab] OR engaged[tiab] OR engagement[tiab] OR retention[tiab] OR retained[tiab] OR linked[tiab] OR linkage[tiab] OR linkages[tiab] OR referral[tiab] OR referred[tiab]) OR (incentives[tiab] OR enablers[tiab] OR “culturally adapted”[tiab] OR “culturally relevant”[tiab]) OR ("Population Surveillance"[mh] OR “prevalence studies”[mh] OR prevalence[tiab]) | 2979110 | 1128377 | 4107487 |
| #2 | Search “Homeless Persons”[mh] OR “Homeless Youth”[mh] OR Homeless[tiab] OR homelessness[tiab] OR shelter*[tiab] OR “transitional housing” [tiab] OR “single room occupancy” [tiab] OR unhoused[tiab] OR “unstably housed” [tiab] OR “unstable housing” [tiab] OR itinerant[tiab] OR “street people”[tiab] OR “street person”[tiab] OR “street youth” [tiab] OR “living rough”[tiab] OR transients[tiab] OR drifters[tiab] OR migrants[tiab] OR vagabonds[tiab] OR hoboes[tiab] OR veterans[tw] | 144288 | 22146 | 166434 |
| #1 | Search “United States”[tiab] “United States”[mh] OR USA[tiab] OR US[tiab] OR Alabama[tiab] OR Alaska[tiab] OR Arizona[tiab] OR Arkansas[tiab] OR California[tiab] OR Colorado[tiab] OR Connecticut[tiab] OR Delaware[tiab] OR Florida[tiab] OR Georgia[tiab] OR Hawaii[tiab] OR Idaho[tiab] OR Illinois[tiab] OR Indiana[tiab] OR Iowa[tiab] OR Kansas[tiab] OR Kentucky[tiab] OR Louisiana[tiab] OR Maine[tiab] OR Maryland[tiab] OR Massachusetts[tiab] OR Michigan[tiab] OR Minnesota[tiab] OR Mississippi[tiab] OR Missouri[tiab] OR Montana[tiab] OR Nebraska[tiab] OR Nevada[tiab] OR “New Hampshire”[tiab] OR “New Jersey”[tiab] OR “New Mexico”[tiab] OR “New York”[tiab] OR “North Carolina”[tiab] OR “North Dakota”[tiab] OR Ohio[tiab] OR Oklahoma[tiab] OR Oregon[tiab] OR Pennsylvania[tiab] OR “Rhode Island”[tiab] OR “South Carolina”[tiab] OR “South Dakota”[tiab] OR Tennessee[tiab] OR Texas[tiab] OR Utah[tiab] OR Vermont[tiab] OR Virginia[tiab] OR Washington[tiab] OR “West Virginia”[tiab] OR Wisconsin[tiab] OR Wyoming[tiab] OR American[tiab] | 940959 | 277816 | 1218775 |

| **Search** | **Embase query** | **Records up to 2016** | **Records 2016-2020** | **TOTAL RECORDS** |
| --- | --- | --- | --- | --- |
| **#5** | **#1 AND #2 AND #3 AND #4** | 1477 | 1783 | **3260** |
| #4 | homeless OR 'homelessness'/exp OR 'homelessness' OR shelter OR shelters OR 'transitional housing' OR 'single room occupancy' OR unhoused OR 'unstably housed' OR 'unstable housing' OR itinerant OR 'street people' OR 'street person' OR 'street youth' OR 'living rough' OR transients OR drifters OR vagabonds OR hoboes OR 'veterans'/exp OR veterans | 204623 | 72715 | 277338 |
| [#3](http://www-ncbi-nlm-nih-gov.ucsf.idm.oclc.org/pubmed/advanced) | 'tuberculosis'/exp OR 'tuberculosis' OR ‘TB’ OR 'ltbi' OR 'hiv'/exp OR 'hiv' OR 'human immunodeficiency virus'/exp OR 'human immunodeficiency virus' OR 'hepatitis b'/exp OR 'hepatitis b' OR 'hepatitis c'/exp OR 'hepatitis c' OR 'hbv'/exp OR 'hbv' OR hcv OR 'hep b' OR 'hep c' OR 'blood-borne' OR 'bloodborne' | 863451 | 225205 | 1088656 |
| #2 | 'mobile health units'/exp OR 'mobile health units' OR screen OR screened OR 'screening'/exp OR 'screening' OR (targeted AND testing) OR program OR 'community based' OR outreach OR recruit OR recruitment OR peer OR 'peer-based' OR 'chain-referral' OR snowball OR 'sampling'/exp OR 'sampling' OR 'time-location' OR 'respondent-driven' OR 'social marketing'/exp OR 'social marketing' OR 'health fair' OR engage OR engaged OR engagement OR retention OR retained OR linked OR linkage OR linkages OR 'referral'/exp OR 'referral' OR referred OR incentives OR enablers OR 'culturally adapted' OR 'culturally relevant' OR surveillance OR 'prevalence'/exp OR 'prevalence' | 4554030 | 6245145 | 10799175 |
| #1 | 'united states'/exp OR 'usa':ab,ti OR 'alabama':ab,ti OR 'alaska':ab,ti OR 'arizona':ab,ti OR 'arkansas':ab,ti OR 'california':ab,ti OR 'colorado':ab,ti OR 'connecticut':ab,ti OR 'delaware':ab,ti OR 'florida':ab,ti OR 'georgia':ab,ti OR 'hawaii':ab,ti OR 'idaho':ab,ti OR 'illinois':ab,ti OR 'indiana':ab,ti OR 'iowa':ab,ti OR 'kansas':ab,ti OR 'kentucky':ab,ti OR 'louisiana':ab,ti OR 'maine':ab,ti OR 'maryland':ab,ti OR 'massachusetts':ab,ti OR 'michigan':ab,ti OR 'minnesota':ab,ti OR 'mississippi':ab,ti OR 'missouri':ab,ti OR 'montana':ab,ti OR 'nebraska':ab,ti OR 'nevada':ab,ti OR 'new hampshire':ab,ti OR 'new jersey':ab,ti OR 'new mexico':ab,ti OR 'new york':ab,ti OR 'north carolina':ab,ti OR 'north dakota':ab,ti OR 'ohio':ab,ti OR 'oklahoma':ab,ti OR 'oregon':ab,ti OR 'pennsylvania':ab,ti OR 'rhode island':ab,ti OR 'south carolina':ab,ti OR 'south dakota':ab,ti OR 'tennessee':ab,ti OR 'texas':ab,ti OR 'utah':ab,ti OR 'vermont':ab,ti OR 'virginia':ab,ti OR 'washington':ab,ti OR 'west virginia':ab,ti OR 'wisconsin':ab,ti OR 'wyoming':ab,ti OR 'american':ab,ti | 2302954 | 3629306 | 5932260 |

| **Search** | **Web of Science query**  **(titles, abstracts and keywords)** | **Records up to 2016** | **Records 2016-2020** | **TOTAL RECORDS** |
| --- | --- | --- | --- | --- |
| **#5** | **#1 AND #2 AND #3 AND #4** | 1000 | 509 | **1509** |
| #4 | homeless OR homelessness OR shelter OR shelters OR “transitional housing” OR “single room occupancy” OR unhoused OR “unstably housed” OR “unstable housing” OR itinerant OR “street people” OR “street person” OR “street youth” OR “living rough” OR transients OR drifters OR vagabonds OR hoboes OR veterans | | | |
| #3 | tuberculosis OR TB OR ltbi OR hiv OR hiv/aids OR “human immunodeficiency virus” OR “hepatitis b” OR “hepatitis c” OR hbv OR hcv OR “hep b” OR “hep c” OR blood-borne OR bloodborne | | | |
| #2 | “mobile health units” OR screen OR screened OR screening OR (targeted AND testing) OR program OR community based OR outreach OR recruit OR recruitment OR peer OR peer-based OR chain-referral OR snowball OR sampling OR time-location OR “respondent-driven” OR “social marketing” OR “health fair” OR engage OR engaged OR engagement OR retention OR retained OR linked OR linkage OR linkages OR referral OR referred OR incentives OR enablers OR “culturally adapted” OR “culturally relevant” OR surveillance OR prevalence | | | |
| #1 | “united states” OR usa OR alabama OR alaska OR arizona OR arkansas OR california OR colorado OR connecticut OR delaware OR florida OR georgia OR hawaii OR idaho OR illinois OR indiana OR iowa OR kansas OR kentucky OR louisiana OR maine OR maryland OR massachusetts OR michigan OR minnesota OR mississippi OR missouri OR montana OR nebraska OR nevada OR “new Hampshire” OR “new jersey” OR “new mexico” OR “new York” OR “north Carolina” OR “north Dakota” OR ohio OR oklahoma OR oregon OR pennsylvania OR “rhode island” OR “south Carolina” OR “south Dakota” OR tennessee OR texas OR utah OR vermont OR virginia OR washington OR “west Virginia” OR wisconsin OR wyoming OR american | | | |

| **Search** | **Cochrane CENTRAL query**  **(titles, abstracts and keywords)** | **Records up to 2016** | **Records 2016-2020** | **TOTAL RECORDS** |
| --- | --- | --- | --- | --- |
| **#5** | **#1 AND #2 AND #3 AND #4** | 56 | 53 | **109** |
| #4 | homeless OR homelessness OR shelter OR shelters OR “transitional housing” OR “single room occupancy” OR unhoused OR “unstably housed” OR “unstable housing” OR itinerant OR “street people” OR “street person” OR “street youth” OR “living rough” OR transients OR drifters OR vagabonds OR hoboes OR veterans | | | |
| #3 | tuberculosis OR TB OR ltbi OR hiv OR hiv/aids OR “human immunodeficiency virus” OR “hepatitis b” OR “hepatitis c” OR hbv OR hcv OR “hep b” OR “hep c” OR blood-borne OR bloodborne | | | |
| #2 | “mobile health units” OR screen OR screened OR screening OR (targeted AND testing) OR program OR community based OR outreach OR recruit OR recruitment OR peer OR peer-based OR chain-referral OR snowball OR sampling OR time-location OR “respondent-driven” OR “social marketing” OR “health fair” OR engage OR engaged OR engagement OR retention OR retained OR linked OR linkage OR linkages OR referral OR referred OR incentives OR enablers OR “culturally adapted” OR “culturally relevant” OR surveillance OR prevalence | | | |
| #1 | “united states” OR usa OR alabama OR alaska OR arizona OR arkansas OR california OR colorado OR connecticut OR delaware OR florida OR georgia OR hawaii OR idaho OR illinois OR indiana OR iowa OR kansas OR kentucky OR louisiana OR maine OR maryland OR massachusetts OR michigan OR minnesota OR mississippi OR missouri OR montana OR nebraska OR nevada OR “new Hampshire” OR “new jersey” OR “new mexico” OR “new York” OR “north Carolina” OR “north Dakota” OR ohio OR oklahoma OR oregon OR pennsylvania OR “rhode island” OR “south Carolina” OR “south Dakota” OR tennessee OR texas OR utah OR vermont OR virginia OR washington OR “west Virginia” OR wisconsin OR wyoming OR american | | | |
