# Supplementary material for: Viral blood-borne infections testing and linkage to care cascade among persons who experience homelessness in the United States: a systematic review and meta-analysis
Source: BMC Public Health. 2022 Jul 26;22:1421. doi: 10.1186/s12889-022-13786-6 (PMC9327172; doi:10.1186/s12889-022-13786-6)
Supplement: Supplementary file 2 — Additional file 2. [file 12889_2022_13786_MOESM2_ESM.docx]

**Additional file 4: articles screened at the full text level**

**Viral blood-borne infections testing and linkage to care cascade among homeless persons in the United States: A systematic review and meta-analysis**

**Contents**

Included (n=24) ……………………………………………………………………………………...…...1

Excluded. Data collection before 1996 cut off (n=1) ……………………………………………………..3

Excluded: Papers could not be retrieved (n=5) …………………………………………….…………….3

Excluded: Do not report required outcomes (n=11) …………………………………………………...….4

Excluded: Not a testing program (n=5) ……………………………………………………………………5

Excluded: Do not report homeless population separately (n=11) ………………………………………….5

Excluded: Homeless population sample size not met (n=3) ……………………………………………….6

Excluded. Duplicative of other study results (n=2) ………………………………………………………..6

**Included (n=24)**

**Peer reviewed articles (n=21)**

1. Grimley DM, Annang L, Lewis I, Smith RW, Aban I, Hooks T, et al. Sexually transmitted infections among urban shelter clients. Sexually transmitted diseases. 2006;33:666-9.
2. Anaya HD, Butler JN, Knapp H, Chan K, Conners EE, Rumanes SF. Implementing an HIV Rapid Testing-Linkage-to-Care Project Among Homeless Individuals in Los Angeles County: A Collaborative Effort Between Federal, County, and City Government. American Journal of Public Health. 2015;105:85-90.
3. Bell DN, Martinez J, Botwinick G, Shaw K, Walker LE, Dodds S, et al. Case finding for HIV-positive youth: a special type of hidden population. The Journal of adolescent health : official publication of the Society for Adolescent Medicine. 2003; 33:10-22.
4. Benitez, T. M., Fernando, S. M., Amini, C., & Saab, S. Geographically Focused Collocated Hepatitis C Screening and Treatment in Los Angeles’s Skid Row. Digestive diseases and sciences. 2020. 65: 3023-3031.
5. Bowles KE, Clark HA, Tai E, Sullivan PS, Song B, Tsang J, et al. Implementing rapid HIV testing in outreach and community settings: Results from an advancing HIV prevention demonstration project conducted in seven U.S. cities. Public Health Reports. 2008;123:78-85.
6. Boyce DE, Tice AD, Ona FV, Akinaka KT, Lusk H. Viral hepatitis in a homeless shelter in Hawai'i. Hawaii medical journal. 2009;68:113-5.
7. Bucher JB, Thomas KM, Guzman D, Riley E, Dela Cruz N, Bangsberg DR. Community-based rapid HIV testing in homeless and marginally housed adults in San Francisco. HIV medicine. 2007;8:28-31.
8. Caton CL, El-Bassel N, Gelman A, Barrow S, Herman D, Hsu E, et al. Rates and correlates of HIV and STI infection among homeless women. AIDS and behavior. 2013;17:856-64.
9. Gelberg L, Robertson MJ, Arangua L, Leake BD, Sumner G, Moe A, et al. Prevalence, distribution, and correlates of hepatitis C virus infection among homeless adults in Los Angeles. Public health reports (Washington, DC : 1974). 2012;127:407-21.
10. Fuster, D., & Gelberg, L. Community screening, identification, and referral to primary care, for hepatitis C, B, and HIV among homeless persons in Los Angeles. Journal of community health. 2019;.44: 1044-1054.
11. Hall CS, Charlebois ED, Hahn JA, Moss AR, Bangsberg DR. Hepatitis C virus infection in San Francisco's HIV-infected urban poor: High prevalence but low treatment rates. Journal of General Internal Medicine. 2004;19:357-65.
12. Hooshyar D, Suris AM, Czarnogorski M, Lepage JP, Bedimo R, North CS. Rapid HIV testing experience at Veterans Affairs North Texas Health Care System's Homeless Stand Downs. AIDS care. 2014;26:95-9.
13. Klinkenberg WD, Caslyn RJ, Morse GA, Yonker RD, McCudden S, Ketema F, et al. Prevalence of human immunodeficiency virus, hepatitis B, and hepatitis C among homeless persons with co-occurring severe mental illness and substance use disorders. Comprehensive Psychiatry. 2003;44:293-302.
14. Magura S, Nwakeze PC, Rosenblum A, Joseph H. Substance misuse and related infectious diseases in a soup kitchen population. Substance Use & Misuse. 2000;35:551-83.
15. Page, K., Yu, M., Cohen, J., Evans, J., Shumway, M., & Riley, E. D. HCV screening in a cohort of HIV infected and uninfected homeless and marginally housed women in San Francisco, California. BMC Public Health.2017;17: 1-9.
16. Robbins JL, Wenger L, Lorvick J, Shiboski C, Kral AH. Health and oral health care needs and health care-seeking behavior among homeless injection drug users in San Francisco. Journal of urban health : bulletin of the New York Academy of Medicine. 2010;87:920-30.
17. Rosenblum A, Nuttbrock L, McQuistion HL, Magura S, Joseph H. Hepatitis C and substance use in a sample of homeless people in New York City. Journal of Addictive Diseases. 2001;20:15-25.
18. Schwarz KB, Garrett B, Alter MJ, Thompson D, Strathdee SA. Seroprevalence of HCV infection in homeless Baltimore families. Journal of health care for the poor and underserved. 2008;19:580-7.
19. Sena AC, Willis SJ, Hilton A, Anderson A, Wohl DA, Hurt CB, et al. Efforts at the Frontlines: Implementing a Hepatitis C Testing and Linkage-to-Care Program at the Local Public Health Level. Public health reports (Washington, DC : 1974). 2016;131 Suppl 2:57-64.
20. Stewart, J., Green, M., Dhanireddy, S., & Golden, M. P066 A mobile clinic model to care for women engaging in exchange sex who are opiate dependent and living unhoused in seattle.2019;
21. Tsu RC, Burm ML, Gilhooly JA, Sells CW. Telephone vs. face-to-face notification of HIV results in high-risk youth. The Journal of adolescent health: official publication of the Society for Adolescent Medicine. 2002;30:154-60.

**Conference abstracts (n=3)**

1. Khalili, M., Powell, J., Naugle, J., Ricco, M., Magee, C., Bush, D., ... & Masson, C. Shelter-based integrated HCV care model is effective in scaling up HCV testing and treatment in homeless clients across two large urban settings. Hepatology.2020; 72:573A-573A.
2. Anaya H, Feld J, Hoang T, Knapp H, Asch S. Implementing an HIV rapid testing intervention for homeless veterans in shelter settings within Los Angeles county. Journal of the International Association of Physicians in AIDS Care. 2010;9:47
3. Preston S, Heaney S, Andrews J. A hepatitis C screening program for the homeless in New Orleans. Journal of General Internal Medicine. 2016;31:S96.

**Excluded. Data collection before 1996 cut off (n=1)**

1. Desai, R. A., Rosenheck, R. A., & Agnello, V. Prevalence of Hepatitis C virus infection in a sample of homeless veterans. Soc Psychiatry Psychiatr Epidemiol. 2003; 38: 396-401.

**Excluded. Papers could not be retrieved (n=5)**

1. Rubin, J. and Dave, S. and Aulakh, H. and Bacchus, A. and Earnest, G. E. & Gager, K. and Tubbs, R. and Zejnullahu, K. and Dulay, M. Improving age-based hepatitis c screening at a veteran’s affairs primary care clinic. Hepatology. 2016; 64(1).
2. Ruiz, G. and Scrudder, K. and Andry, C. and Miller, N. and Perkins, E. & Burner, E. Improvements to electronic medical record result in increased hepatitis C screening and treatment. Academic Emergency Medicine. 2019; 26: S241-S242.
3. Preston, S. and Heaney, S. and Andrews, J. A hepatitis C screening program for the homeless in New Orleans. Journal of General Internal Medicine. 2016; 3(2): S96.
4. Jones, A. T. and McGonigle, K. M. and Carley, T. F. and Nix, L. and Kim, M. M., Mallya, S. G., Coyne, M. O., Lee, F., Kanter, J. M., Kissinger, P. & Rajan, L. Enabling healthcare access for hepatitis C through community-based screening and linkage to care. American Journal of Tropical Medicine and Hygiene. 2018;99(4).
5. Ramers, C. B. and Rojas, S. A. and Constantino, S. and Asmus, L. and Cavanaugh, C., Khasira, M. & Frenette, C. T. Eliminating hepatitis c in the medical home: Hepatology, GI, and ID collaboration to build primary care capacity for treatment. Hepatology. 2018;68: 465A - 466A.

**Excluded. Do not report required outcomes (n= 11)**

1. Rapid HIV testing popular with Chicago CBO clients. Testing has found 2% positive rate. AIDS Alert. 2005; 20: 22-23.
2. Massachusetts project cuts a wide swath of care. Nontraditional testing sites is main focus. AIDS Alert*.* 2005; 20: 18-19.
3. Beech, B. M., Myers, L., & Beech, D. J. Hepatitis B and C infections among homeless adolescents. Family & community health*.*2002; *25*:28-36.
4. Knapp, H., Anaya, H. D., & Feld, J. E. Expanding HIV rapid testing via point-of-care paraprofessionals. Int J STD AIDS*.* 2008*;*19: 629-632.
5. Myers, T., Muvva, R., Nganga-good, C., Ndirangu, H., & Fields, N. Taking HIV testing to those at the highest risk: Testing in non-traditional venues through outreach in Baltimore, Maryland. Sexually Transmitted Diseases. 2014; 41:S12.
6. Nyamathi, A. M., Marlow, E., Branson, C., Marfisee, M., & Nandy, K. Hepatitis A/B vaccine completion among homeless adults with history of incarceration. J Forensic Nurs. 2012;8: 13-22.
7. Robertson, M. J., Clark, R. A., Charlebois, E. D., Tulsky, J., Long, H. L., Bangsberg, D. R., & Moss, A. R. HIV seroprevalence among homeless and marginally housed adults in San Francisco. American Journal of Public Health. 2004; 94:1207-1217.
8. Winkelstein, E. R., Edlin, B. R., Szott, K., Shu, M. A., McKnight, C., DesJarlais, D. C., . . . Haberlen, E. The SWAN Project: Integrating research and service with a cohort of young people who use drugs at risk for hepatitis C in New York City. Suchtmedizin in Forschung und Praxis. 2013;15: 238.
9. Hanlon, P., Yeoman, L., Gibson, L., Esiovwa, R., Williamson, A. E., Mair, F. S., & Lowrie, R. A systematic review of interventions by healthcare professionals to improve management of non-communicable diseases and communicable diseases requiring long-term care in adults who are homeless. BMJ open. 2018; 8: e020161.
10. Hodges, J., Reyes, J., Campbell, J., Klein, W., & Wurcel, A. Successful implementation of a shared medical appointment model for hepatitis C treatment at a community health center. Journal of community health. 2019; 44: 169-171.
11. Bakr, O., Gelberg, L., Cowan, B., Seragaki, S., Youn, S., Kawamoto, J. K., ... & Bhattacharya, D. 798-Treating Hepatitis C in the Homeless Patient-Aligned Care Team (HPACT) at the Greater Los Angeles VA–A Pilot Study. Gastroenterology. 2018; 154: S-1105.

**Excluded. Not a testing program (n=5)**

1. Hodges, J., Reyes, J., Campbell, J., Klein, W., & Wurcel, A. Successful implementation of a shared medical appointment model for hepatitis C treatment at a community health center. Journal of community health. 2019; 44: 169-171.
2. Hawk, M., Maulsby, C., Enobun, B., & Kinsky, S. HIV Treatment Cascade by Housing Status at Enrollment: Results from a Retention in Care Cohort. AIDS and Behavior. 2019; 23: 765-775.
3. Lubega, S., Agbim, U., Surjadi, M., Mahoney, M., & Khalili, M. Formal hepatitis C education enhances HCV care coordination, expedites HCV treatment and improves antiviral response. Liver International. 2013; 33:999-1007.
4. Desai, M. M., & Rosenheck, R. A. HIV testing and receipt of test results among homeless persons with serious mental illness. American Journal of Psychiatry. 2004; 161: 2287-2294.
5. Norton, B. L., Voils, C. I., Timberlake, S. H., Hecker, E. J., Goswami, N. D., Huffman, K. M., . . . Stout, J. E. Community-based HCV screening: knowledge and attitudes in a high risk urban population. BMC Infect Dis. 2014; 14: 74.

**Excluded. Do not report homeless sub-population separately (n=11)**

1. Camacho-Gonzalez, A. F., Gillespie, S. E., Thomas-Seaton, L., Frieson, K., Hussen, S. A., Murray, A., ... & Chakraborty, R. The Metropolitan Atlanta community adolescent rapid testing initiative study: closing the gaps in HIV care among youth in Atlanta, Georgia, USA. AIDS (London, England). 2017;31: S267.
2. Lubega, S., Agbim, U., Surjadi, M., Mahoney, M., & Khalili, M. Formal hepatitis C education enhances HCV care coordination, expedites HCV treatment and improves antiviral response. Liver International. 2013; 33: 999-1007.
3. Zucker, D. M., Choi, J., & Gallagher, E. R. Mobile outreach strategies for screening hepatitis and HIV in high-risk populations. Public Health Nurs. 2012; 29:27-35
4. Coyle, C., Viner, K., Hughes, E., Kwakwa, H., Zibbell, J. E., Vellozzi, C., & Holtzman, D. Identification and Linkage to Care of HCV-Infected Persons in Five Health Centers - Philadelphia, Pennsylvania, 2012-2014. MMWR Morb Mortal Wkly Rep. 2015; 64: 459-463.
5. Ford, M., Johnson, N., Desai, P., Rude, E. J., & Laraque, F. From care to a cure: Improving the hepatitis C care cascade through patient navigation in the check hep C program in New York City. Hepatology. 2015;62: 1086A.
6. Ford, M., Jordan, A., Rude, E. J., Johnson, N., Hagan, H., Laraque, F., & Varma, J. K. Check hep C: A community-based approach to hepatitis C diagnosis in high-risk populations. Hepatology. 2014; 60: 894A.
7. Plax, K., Garbutt, J., & Kaushik, G. N. HIV and Sexually Transmitted Infection Testing Among High-Risk Youths: Supporting Positive Opportunities With Teens (SPOT) Youth Center. American Journal of Public Health. 2015; 105: 1394-1398.
8. Surratt, H. L., & Inciardi, J. A. HIV risk, seropositivity and predictors of infection among homeless and non-homeless women sex workers in Miami, Florida, USA. AIDS Care. 2004; 16:594-604.
9. Trooskin, S. B., Poceta, J., Towey, C. M., Yolken, A., Rose, J. S., Luqman, N. L., . . . Nunn, A. S. Results from a Geographically Focused, Community-Based HCV Screening, Linkage-to-Care and Patient Navigation Program. J Gen Intern Med. 2015; 30:950-957.
10. Zetola, N. M., Grijalva, C. G., Gertler, S., Hare, C. B., Kaplan, B., Dowling, T., . . . Klausner, J. D. Simplifying consent for HIV testing is associated with an increase in HIV testing and case detection in highest risk groups, San Francisco January 2003-June 2007. PLoS ONE. 2008; 3
11. Zucker, D. M. Mobile outreach for hepatitis C and HIV in Western Massachusetts. Gastroenterology. 2009; 136: A833.

**Excluded. Homeless population sample size not met (n=3)**

1. Coyle, C., Moorman, A. C., Bartholomew, T., Klein, G., Kwakwa, H., Mehta, S. H., & Holtzman, D. The hepatitis C virus care continuum: linkage to hepatitis C virus care and treatment among patients at an urban health network, Philadelphia, PA. Hepatology. 2019; 70: 476-486.
2. Burton, M. J., Voluse, A. C., & Anthony, V. Integrating comprehensive hepatitis C virus care within a residential substance use disorder treatment program. Journal of substance abuse treatment. 2019; 98: 9-14.
3. Blackwell, J. A., Rodgers, J. B., Franco, R. A., Cofield, S. S., Walter, L. A., Galbraith, J. W., & Hess, E. P. Predictors of linkage to care for a nontargeted emergency department hepatitis C screening program. The American journal of emergency medicine. 2020; 38: 1396-1401.

**Excluded. Duplicative of other study results (n=2)**

1. Stein, J. A., Andersen, R. M., Robertson, M., & Gelberg, L. Impact of hepatitis B and C infection on health services utilization in homeless adults: A test of the Gelberg-Andersen behavioral model for vulnerable populations. Health Psychology. 2012; 31: 20-30.
2. Strehlow, A. J., Robertson, M. J., Zerger, S., Rongey, C., Arangua, L., Farrell, E., . . . Gelberg, L. Hepatitis C among clients of health care for the homeless primary care clinics. Journal of Health Care for the Poor and Underserved. 2012; 23: 811-833.
